# Influence of light intensity, fertilizing and season on the cirsiliol content, a chemical marker of *Leonotis nepetifolia* (Lamiaceae)

**DOI:** 10.7717/peerj.6187

**Published:** 2019-01-15

**Authors:** Ana Paula de Oliveira, Ivanildo Viana Borges, Emanuella Chiara Valença Pereira, Thiala Alves Feitosa, Raira Feitosa dos Santos, Raimundo Gonçalves de Oliveira-Junior, Larissa Araújo Rolim, Lucas Gustavo Ferreira Cordeiro Viana, Luciano Augusto de Araújo Ribeiro, Alan Diego da Conceição Santos, Pedro José Rolim-Neto, Jackson Roberto Guedes da Silva Almeida

**Affiliations:** 1 Center for Studies and Research of Medicinal Plants, Federal University of Vale do São Francisco, Petrolina, Pernambuco, Brazil; 2 Pharmacy, Federal University of Pernambuco, Recife, Pernambuco, Brazil

**Keywords:** Lamiaceae, Flavonoids, Caatinga, Cirsiliol

## Abstract

**Background:**

*Leonotis nepetifolia* (Family Lamiaceae) is a medicinal plant from which the flavonoid cirsiliol with sedative, hypnotic, anti-inflammatory and cytotoxic activity has been extracted.

**Methods:**

Seedlings were cultivated under different levels of shade in native or fertilized modes. The content of cirsiliol was measured monthly by high-performance liquid chromatography and the total phenolic content by the Folin-Ciocalteu method. Monitoring of growth was carried out with the weekly measurement of height until the stabilization of growth.

**Results:**

The application of fertilizing and/or shading does not alter significantly the cirsiliol content. However, this content varies throughout the year, reaching the peak production in the summer, independently of the treatment applied. This same profile, with production in the summer, was also verified for phenolic compounds, reaching 58.15 ± 9.35 mg of equivalents of gallic acid per g of extract in the summer, content 1.84 times greater than the content verified in winter (31.56 ± 4.09 mg of gallic acid/g of extract). Although shading and fertilizing had no effect on cirsiliol content, the results also showed a positive influence on the height and biomass of the plant, which can causes a higher yield of extractable material.

**Discussion:**

Biotic and abiotic stresses are able to increase or decrease the production of secondary metabolites, including phenolic compounds in medicinal plants and, as the stress response is peculiar to each species, cultivation studies become necessary. The present study reports by the first time the influence of shading, fertilizing and seasons in cirsiliol content in *L. nepetifolia*. Among analyzed variables, the seasons showed a larger influence in expression of cirsiliol and among seasons, our results showed that the summer is the ideal season for collections. In summer, the photoperiod is larger than in other seasons of the year and due to that, the plants need greater protection against the long photoperiod. For this, the plants increase the production of phenolic compounds as observed in this study. Although they do not influence the production of cirsiliol, the shading and nutrients in soil favor growth and leaf area of several plants, explaining, thus, the higher height and biomass obtained.

## Introduction

*Leonotis nepetifolia* (Family Lamiaceae) with pantropical distribution is known in Brazil as “Cordão de São Francisco” (Cruz et al., 2011). Previous studies carried out with extracts of this species revealed the cytotoxic potential against human tumor cell lines MCF7 (breast), Hep2 (human epithelial type 2), SF295 (human brain), OVCAR8 (ovarian) and HCT116 (human colorectal) (Veerabadran et al., 2013; Oliveira et al., 2016). A previous study carried out by our research group provided the identification that the flavonoid cirsiliol is one of the compounds responsible for the cytotoxic activity observed in this plant (Oliveira et al., 2016).

Cirsiliol (3′,4′,5-trihydroxy-6,7-dimethoxyflavone) is a flavone, found in *Leonotis nepetifolia* and several genera of the Lamiaceae family (Viola et al., 1997; Bai et al., 2010; Li et al., 2012) has shown sedative, hypnotic, anti-inflammatory and cytotoxic activity. Besides, this compound showed high selectivity against tumor cells, a goal sought by many cancer researchers (Veerabadran et al., 2013; Rathee et al., 2012). As cirsiliol, other compounds belonging to the flavonoid class are found in plants and present important biological functions, including such potentials as antiviral, antibacterial, estrogenic, anti-obesity, antioxidant, cardioprotector and reducer of platelet aggregation (Maciel et al., 2011; Oliveira et al., 2011; Wang, Chen & Yu, 2011).

Although innumerable biological and pharmacological potentials of flavonoids have been proven, they are produced in low amounts in plants. In plants, the synthesis and accumulation of these compounds are directly connected with defense mechanism against biotic and abiotic stress-causing agents and the adaptation process to climatic changes exhibited by environment (Wang, Chen & Yu, 2011).

Among the biotic and abiotic factors, light and fertilizing are considered as the main stress-causing agents (Souza et al., 2007).

Studies have shown that changes in intensity, composition and light exposure time, modify the production and accumulation of flavonoids in various plants; they have also showed that the response to modifications is peculiar to each species investigated (Morales et al., 2011; Løvdal et al., 2010; Stoffyn et al., 2012; Barnes et al., 2016; Deng et al., 2017). Regarding fertilization, several authors ensure that the accumulation of flavonoids and other phenolic compounds is directly related to the fertilizing deficiency since gene expression for production and accumulation of these compounds is stimulated by the soil nutritional deficiency (Løvdal et al., 2010; Labart et al., 2012; Silva et al., 2015; Dixon & Paiva, 1995).

The development of methods that increase the production of flavonoids, as well as others secondary metabolites in medicinal plants, with minimal cultivation and organic agricultural practices are necessary and frequently recommended (Marques et al., 2018). For this, one needs to understand how a particular crop responds to applied agronomic treatments. Thus, this study aims to verify the effect of shading levels and fertilization on the production of the flavonoid cirsiliol in *L. nepetifolia*, and still try to find out the optimal conditions of cultivation that provide high levels of cirsiliol and biomass favorable for extraction.

## Material and Methods

### Chemical and standards

Cirsiliol (≥95%, UV, HPLC), Folin-Cioucalteu’s phenol reagent and sodium carbonate were purchased from Sigma-Aldrich (St. Louis, MO, USA). Gallic acid was purchased from Vetec (São Paulo, BR). HPLC-grade solvents were purchased from Tedia Brasil (Rio de Janeiro, BR).

### Obtaining and cultivation of seedling

*L. nepetifolia* seeds were sown in a sterilized substrate prepared with a mixture of sand and earthworm humus (4:1) on plastic trays. The trays were irrigated once a day and kept at 25 °C in a greenhouse with 12–12 h light-dark cycle (Santos, Morais & Matos, 2004). After 30 days, plants were transplanted as seedlings for buckets (18 dm^3^) with treatments: unfertilized soil (native soil) or fertilized soil (earthworm humus with native soil (1:1)). The treatments were coded as US and FS, respectively. The seedlings were distributed in nurseries and protected with polypropylene net of black color, presenting the following percentages: 0% (outdoor), 30, 50 and 70% of retention of the solar radiation flow and water once a day (1 dm^3^). During the experimental period, the response of plants to treatments applied for expression of cirsiliol in a given month and among the different months of study was evaluated. Plant height and the content of total phenolic compounds were also evaluated. The studies were carried out in the urban zone of Petrolina (Pernambuco, Brazil) and the experimental design was randomized with 5 plants for each treatment.

### Extraction

After 30 days of transplant, leaves of each sample were collected. The material plant was dried at 40 °C and the powdered material (200 mg) was extracted using ethanol 95% with a solvent drug in the ratio 1:100 at 120 rpm for 2 h at room temperature. The solvent was evaporated in an oven with air circulation at 40 °C. This process was repeated every 30 days.

### HPLC-DAD analysis

Analysis of the concentration of the flavonoid cirsiliol was carried out with high-performance liquid chromatography on a Shimadzu^®^ HPLC apparatus, LC-20AT model, series 02403 equipped with LC-solutions^®^ software, auto-sampler, diode array detector and guard column (12.6 × 4.6 mm i.d., 5.0 µm particle size; Agilent, Santa Clara, CA, USA). The quantification was carried out using a curve calibration made with solutions of the flavonoid in different concentrations (*y* = 101662.45x + 6859.98, *r*^2^ = 0.9958) and the compounds were separated at Zorbax Eclipse Plus C_18_ column (250 × 4.6 mm i.d., 5.0 µm particle size; Agilent, Santa Clara, CA, USA). A binary gradient solution was performed with solvent A (acetic acid 2%) and solvent B (90% methanol, 5% acetic acid and 5% water), delivered at a flow ratio of 0.6 mL.min^−1^ as follows: 0–20 min 25% B; 20–40 min 100% B; 40–60 min 25% B to return to initial conditions. The injection volume of extracts and standard were 20 µL and the analyses were conducted with three and five replicates for both the standard and extracts, respectively.

### Total phenolic compounds

Total phenolic content was assayed using Folin-Ciocalteu reagent based on the method reported by Slinkard & Singleton, (1977). The absorbance was measured at 765 nm using an Even^®^ UV-Vis spectrophotometer. A curve of standard gallic acid (*y* = 0.0013x + 0.0153, *r*^2^ = 0.9989) with range 50–1,000 mg.mL^−1^ was obtained under the same conditions as the samples and the content of total phenolic compound was expressed as mg of gallic acid equivalent to gram of extracts.

### Plant height

The height of each plant was monitored weekly until the stabilization of the growth, measuring the height of the basis to the apex of the central stem. The results were expressed as mean of cm ± standard deviation.

### Statistical analysis

The Gaussian distribution of the data was tested using D’Agostino and Pearson omnibus normality tests. Presence of outliers was verified with Brown-Forsythe and Bartlett’s tests. Analysis of changes caused by treatments under the same packaging was performed using Test-t. The influences of shading × treatments (US and FS) and month × treatments (US and FS) were developed using analysis of variance (two-way anova) followed by Tukey’s as post-test. All analyses were performed using *GraphPad Prism 6.0* software.

## Results

### Cirsiliol content

The quantification of cirsiliol using the high-performance liquid chromatography showed that in the leaves of *L. nepetifolia,* the values ranged between 1.03 ± 0.07 µg/mg of extract as minimum and 8.85 ± 1.24 µg/mg of extract as maximum over the experimental period.

For each month of the study, our results showed that for the same nursery and in the most cases, the cirsiliol content in plants grown in fertilized soil was in a slightly lower concentration than that presented by individuals cultivated on unfertilized soil (Table 1). However, the two-way analysis of variance confronting the factors shading × fertilization showed that these factors did not alter significantly the expression of flavonoid cirsiliol by the plant.

**Table 1:** Influence of fertilizing in expression of cirsiliol during experimental time. Comparison performed using Student test-*t* between fertilized soil and unfertilized soil; Values expressed as media of µg/mg ± standard deviation.

**Season**	**Month**	**Outdoor**			**30**			**50**			**70**		
		**US**	**FS**	***log p***	**US**	**FS**	***log p***	**US**	**FS**	***log p***	**US**	**FS**	***log p***
Winter	Aug-2015	–	–	nd	2.24 ± 0.91	3.94 ± 1.11	*p* = 0.0144	3.22 ± 0.91	2.00 ± 0.72	*p* = 0.0300	1.45 ± 0.27	1.03 ± 0.07	*p* = 0.0316
Spring	Sep-2015	3.49 ± 0.26	1.1 ± 0.19	*p < 0.0001*	1.94 ± 0.75	1.22 ± 0.42	ns	2.28 ± 0.26	1.28 ± 0.25	*p < 0.0008*	1.66 ± 0.23	1.11 ± 0.42	*p* = 0.0204
Spring	Oct-2015	6.07 ± 1.18	5.75 ± 0.43	ns	2.90 ± 0.17	2.74 ± 0.84	ns	4.79 ± 1.33	3.74 ± 0.93	ns	2.96 ± 0.32	2.21 ± 0.66	*p* = 0.0258
Spring	Nov-2015	–	–	nd	3.50 ± 0.67	3.62 ± 1.54	ns	3.33 ± 1.05	4.29 ± 1.19	ns	3.20 ± 0.24	4.69 ± 1.22	ns
Summer	Jan-2016	2.56 ± 0.46	2.81 ± 1.05	ns	4.40 ± 0.56	3.74 ± 1.14	ns	3.83 ± 1.15	5.91 ± 1.01	*p* = 0.0313	4.37 ± 0.38	5.40 ± 1.76	ns
Summer	Feb- 2016	8.85 ± 1.24	6.77 ± 0.53	*p* = 0.0277	7.57 ± 1.46	6.92 ± 1.30	ns	8.50 ± 1.56	7.01 ± 2.11	ns	8.33 ± 0.99	5.64 ± 0.91	*p* = 0.0011
Summer	Mar-2016	5.96 ± 2.86	8.50 ± 1.50	ns	8.44 ± 1.96	6.19 ± 3.30	ns	7.41 ± 0.55	4.71 ± 0.54	*p* = 0.0060	8.73 ± 1.82	5.00 ± 0.53	*p* = 0.0051
Autumn	Apr-016	2.42 ± 0.19	3.59 ± 1.04	ns	4.29 ± 0.36	3.32 ± 1.46	ns	3.75 ± 0.28	2.39 ± 0.40	*p* = 0.0303	8.23 ± 0.62	4.47 ± 2.59	ns
Autumn	May-2016	6.30 ± 0.08	5.33 ± 2.11	ns	5.63 ± 1.56	6.17 ± 1.71	ns	4.12 ± 0.15	4.66 ± 0.17	*p* = 0,0385	3.41 ± 0.16	6.80 ± 0.31	*p* = 0.0025
Winter	Jul-2016	2.75 ± 0.02	1.62 ± 0.61	ns	2.59 ± 0.35	2.60 ± 1.58	ns	3.14 ± 0.22	1.01 ± 0.72	ns	2.22 ± 0.61	0.37 ± 0.07	*p* = 0.0006

**Notes.**

nd not detected ns not significant US unfertilized soil FS fertilized soil with 50% of earthworm humus outdoors plants grown without shading 30 plants grown with 30% shading 50 plants grown with 50% shading 70 plants grown with 70% shading

Data considered with significant difference when *p* < 0.05.

**Figure 1:**
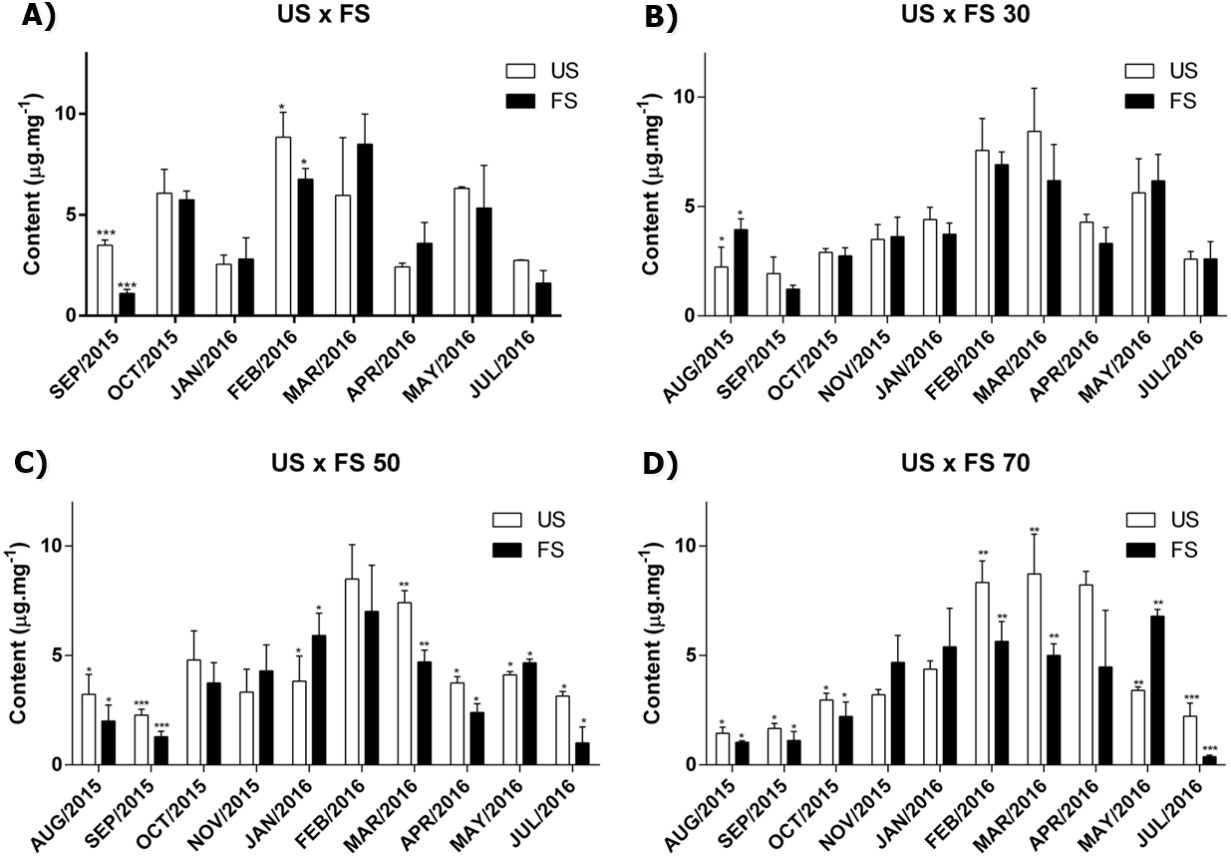
Content of flavonoid cirsiliol in the leaves of *L. nepetifolia* per treatment and months. (A) US × FS; (B) US × FS 30; (C) US × FS 50; (D) US × FS 70. Values expressed as µg.mg^−1^ of crude ethanol extract. US, unfertilized soil; FS, fertilized soil with 50% of earthworm humus; US × FS: plants. Bars with the same symbol have difference statistically significant using *Student test-t*. The difference was statistically significant when *p* < 0.005.

As we can see in Fig. 1, the production of cirsiliol varies during the year and for all treatments, the month collection factor was the main modifying agent in the flavonoid cirsiliol content, representing 76.50, 67.75, 66.86 and 68.21% of observed variations in (two-way ANOVA) analyses, followed by Tukey’s post-test for outdoors, 30, 50 and 70% of shading, respectively. These conclusions were obtained confronting the factors month × fertilization for each shading applied.

### Influence of season

The grouping of results according to the season allowed us to observe that a peak of expression of cirsiliol was achieved in the summer for all treatments (Table S2). This same profile was verified in the quantification of total phenols using the Folin-Ciocalteu method, where a larger content of total phenolic compounds (58.15 ± 9.35 mg of gallic acid per gram of extract) was achieved in the summer followed by autumn (57.08 ± 5.63), spring (32.68 ± 17.78) and winter 31.56 ± 4.09.

### Plant height

**Figure 2:**
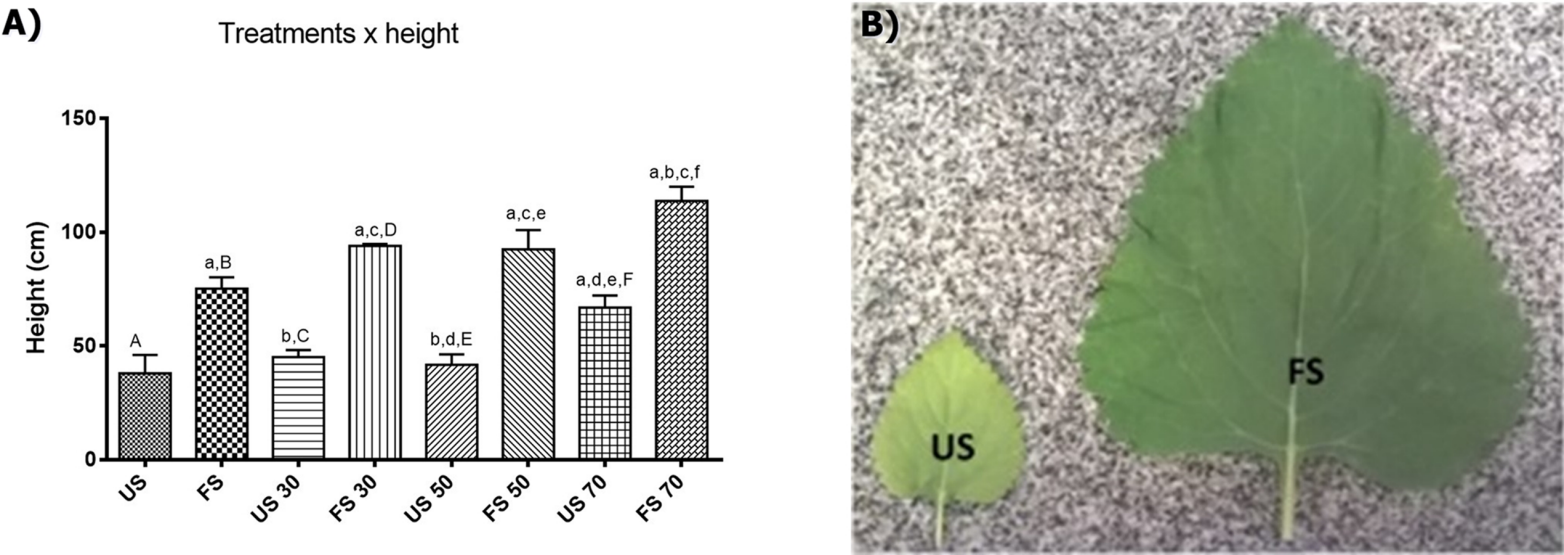
Height of plants per treatment applied (A) and leaves per treatment in soil (B). Values expressed as cm ± SD. US, plants grown outdoors in unfertilized soil; FS, plants grown outdoors in fertilized soil with 50% of earthworm humus; US 30, plants. Groups with lowercase letters present statistically significant difference when compared to their respective capital letters using one-way ANOVA and Tukey as post-test; the difference was considered significant when *p* < 0.05.

In *L. nepetifolia*, the individual growths were stabilized after 45 days of transplantation. The minimum height (38.00 ± 16.15 cm) was achieved for individuals grown in unfertilized soil and outdoors and the maximum height, was achieved for individuals grown in fertilized soil with 70% shading (113.8 ± 13.95 cm), a value three times higher than the one observed for the plants with lower height (Table S2). The results obtained still suggest a positive correlation among the variables shading, fertilizing and height (Fig. 2).

To know the contribution of each factor (shading and fertilizing) in the height and biomass of plants, the data were analyzed through the two-way analysis of variance correlating the height with the factors shading and fertilizing. The results showed that both factors have a positive influence in the height and biomass of plants, and also that light contributes with 19.32% and the fertilization with 65.49% of the variance observed.

## Discussion

Biotic and abiotic stresses are able to increase or decrease the production of secondary metabolites, including phenolic compounds, in medicinal plants.

Phenolic compounds, such as flavonoids and hydroxyl cinnamic acids, attenuate the UV radiation, absorbing harmful UV and transferring the photosynthetically active radiation to mesophyll active cells (Morales et al., 2011; Barnes et al., 2016). Based on this information, the influence of light in cirsiliol content aroused our curiosity once that for plants grown outdoors, a higher content of cirsiliol was expected. The reason why our results were related to the production of phenolic compounds is because of the regulation by specific wavelengths (UV-B radiation), which tend to increase the synthesis and accumulation of phenolic compounds (Jenkins, 2014; Barnes et al., 2016; Deng et al., 2017) and thus, our results showed that the variation of shading applied did not provide selectivity for the wavelengths that was capable to alter the synthesis and accumulation of flavonoid cirsiliol.

Considering the treatment applied to soil, in the same nursery, the results showed a slight decrease in the cirsiliol content for individuals grown in fertilized soil. The results are related to the high availability and equilibrium of nutrients provided by organic fertilization that reduces the conditions of nutritional stress and the production of secondary metabolites (Silva et al., 2015). Organic fertilizers are rich in nitrogen and several authors have suggested the negative correlation among their excess in soil and phenolic compound content (Løvdal et al., 2010; Labart et al., 2012; Silva et al., 2015; Dixon & Paiva, 1995; Akula & Ravishankar, 2011). In order to analyze the influence of nutrients, more specifically nitrogen in *L. nepetifolia,* the earthworm compost was chosen because of its high content and availability of this nutrient (Oliveira et al., 2001).

As observed in our results for flavonoid cirsiliol, a study developed by Silva et al. (2015) showed that the addition of organic fertilizer to *Ageratum conyzoides* L. decreased the content of total flavonoids. Løvdal et al. (2010) observed that the increase of nitrogen content has a negative correlation with the levels of phenolic compounds in the leaves of tomato (*Solanum lycopersicum*, *cv*. *Suzanne*). Labart et al. (2012) in another tomato culture (*Solanum lycopersicum*, *cv*. *Pixie*, F1 Hybrid) observed that the content of leaf phenolic concentrations was strongly enhanced with N limitation. However, in a study developed by Chrysargyris, Panayiotou & Tzortzakis (2016) in *Lavandula angustifolia* Mill, species also belonging to the Lamiaceae family, the content of flavonoids and phenolic compounds increased with high nitrogen concentrations. These controversies among the results found by different authors in different cultures reaffirm the need for studies aimed at each medicinal species to be cultivated.

As seen previously, the production of cirsiliol varies during the year and though there is a tendency that fertilizing reduces the cirsiliol content, the two-way analyses of variance (ANOVA) showed us that the factor fertilizing represents only 0.54, 0.44, 2.94 and 2.16% for outdoors, 30, 50 and 70% shading, respectively, of the variation observed, what makes it negligible.

For a better understanding of the production profile of the flavonoid cirsiliol exhibited by *L. nepetifolia* in this study, the results were grouped by season and all the treatments showed the same profile, with a peak production of flavonoid cirsiliol in the summer. Akula & Ravishankar (2011) to affirm that the plants are extremely sensitive to climatic changes and many authors have proved it. In *Phyllanthus amarus,* species belonging to Lamiaceae family, the content of phenolic compounds also increased in the summer (Gehlot & Kasera, 2013). Macedo et al. (2013) found results similar to ours in a study developed with *Davilla rugose* Poir. In this species cultivated in Brazil, the content of total flavonoids and tannins was also higher in summer.

In summer, the photoperiod is larger than in other seasons of the year (Branco, 2017) and due to that, the plants need a greater protection against the long photoperiod. This higher photoprotection is obtained through a higher synthesis of phenolic compounds as observed for *L. nepetifolia* and for other species mentioned.

Light and fertilizing are environmental factors influencing also upon the growth and development of plants. Each species responds in a peculiar way to modifications of light intensities, while nutrient tends to favor plant development (Oliveira et al., 2001; Souza et al., 2007). Given the positive influence of soil nutrients in the promotion of greater plant development, our results are according that expected.

Our results corroborating still other researches that showed the increased height and foliar area promoted by shading. In alecrim-pimenta (*Lippia sidoides* Cham.), the cultivation with 25% shading increased 1.45 times the size of plants and 1.1 times the foliar area (Souza et al., 2007); in a study developed with *E. pseudowushanense*, the seedlings grown under relatively low light intensities had larger leaves and larger biomass compared with those grown under high light intensities (Pan & Guo, 2016). In light absence, the plants tend to improve the foliar area to increase the uptake of solar radiation (Souza et al., 2007) which explain the results found in *L. sidoides*, *E. pseudowushanense* and in *L. nepetifolia* in this study.

## Conclusions

In summary, our results demonstrated that the photoperiod is the main factor able to increase the content of total phenolic compounds and the cirsiliol content. Our study also demonstrated that the treatment with fertilization and shading provided a higher height and consequently a higher biomass for *L. nepetifolia* species. Hence, studies that relate the time of exposure to solar radiation and the content of the flavonoid cirsiliol are necessary for a higher optimization of the production of this compound by the species under study.

##  Supplemental Information

10.7717/peerj.6187/supp-1 Supplemental Information 1 Tables S1 and S2

10.7717/peerj.6187/supp-2 Data S1 Raw data

## Additional Information and Declarations

### Data Availability

The following information was supplied regarding data availability: Raw data is available in the Supplemental Materials.
