# Supplementary material for: Influence of light intensity, fertilizing and season on the cirsiliol content, a chemical marker of Leonotis nepetifolia (Lamiaceae)
Source: PeerJ. 2019 Jan 15;7:e6187. doi: 10.7717/peerj.6187 (PMC6338097; doi:10.7717/peerj.6187)
Supplement: Supplemental Information 1 [file peerj-07-6187-s001.docx]

**Influence of light intensity, fertilizing and season on the cirsiliol content, a chemical marker of *Leonotis nepetifolia* (Lamiaceae)**

**Supplementary material**

Table S1 **Content of cirsiliol per treatment and season**. Mean of values of cirsiliol content expressed as μg.mg-1 of extracts; US: unfertilized soil; FS: fertilized soil with 50% of earthworm humus; 0: plants grown without shading; 30: plants grown with 30% shading; 50: plants grown with 50% shading; 70: plants grown with 70% shading; Groups with lowercase letters present statistically significant difference when compared to their respective capital letters using two-way ANOVA and Tukey as post-test; the difference was considered significant when p < 0.05.

| **Treatment** | **Winter** | **Spring** | **Summer** | **Autumn** |
| --- | --- | --- | --- | --- |
| US 0 | 2.75 ± 0.01 | 5.04 ± 1.65 | 5.78 ± 3.19 | 4.37 ± 2.25 |
| FS 0 | 1.62 ± 0.61 | 2.66 ± 2.41 ^A^ | 6.43 ± 2.55^a^ | 4.46 ± 1.77 |
| US 30 | 2.39 ± 0.78^A^ | 2.68 ± 1.07^b^ | 6.97 ± 2.22ª^,B^ | 4.74 ± 1.02ª^,b^ |
| FS 30 | 3.15 ± 1.39^a^ | 2.36 ± 1.30^a^ | 5.57 ± 2.35^A^ | 4.27 ± 2.01 |
| US 50 | 2.37 ± 1.33^a^ | 3.47 ± 1.40^a^ | 6.85 ± 2.36^A^ | 3.93 ± 0.28^a^ |
| FS 50 | 1.56 ± 0.85^a^ | 3.05 ± 1.66^a^ | 6.06 ± 1.65^A^ | 3.53 ± 1.34^a^ |
| US 70 | 1.93 ± 0.62^A^ | 2.46 ± 0.75 | 6.90 ± 2.29ª^,b^ | 5.82 ± 2.81 |
| FS 70 | 0.66 ± 0.36^A^ | 2.64 ± 1.68^ª,B^ | 5.37 ± 1.09ª^,b^ | 5.14 ± 2.40ª^,b^ |

Table S2 **Height of plants per treatment**. Values in cm expressed as media ± standard deviation; US: unfertilized soil; FS: fertilized soil with 50% of earthworm humus; 0: plants grown without shading; 30: plants grown with 30% shading; 50: plants grown with 50% shading; 70: plants grown with 70% shading; Groups with lowercase letters present statistically significant difference when compared to their respective capital letters using two-way ANOVA and Tukey as post-test; the difference was considered significant when p < 0.05.

|  | **0** | **30** | **50** | **70** |
| --- | --- | --- | --- | --- |
| **US** | 38.00 ± 16.15^A^ | 45.20 ± 6.76^b,C^ | 41.75 ± 9.22^b,d,E^ | 67.00 ± 11.73^a,d,c,F^ |
| **FS** | 75.25 ± 9.95^a,B^ | 94.00 ± 1.87^a,c,D^ | 92.50 ± 16.90^a,c,e^ | 113.8 ± 13.95^a,b,c,f^ |
